# Prospective multicenter non-randomized controlled study on intraosseous stability and healing period for dental implants in the posterior region

**DOI:** 10.1186/s40729-018-0122-x

**Published:** 2018-03-29

**Authors:** Shinya Homma, Yasushi Makabe, Takuya Sakai, Kenzou Morinaga, Satoru Yokoue, Hirofumi Kido, Yasutomo Yajima

**Affiliations:** 1 Department of Oral and Maxillofacial Implantology, Tokyo Dental College, 2-9-18 Misaki-cho, Chiyoda-ku, Tokyo, 101-0061 Japan; 2 Section of Oral Implantology, Department of Oral Rehabilitation, Fukuoka Dental College, 2-15-1 Tamura, Sawara-ku, Fukuoka-City, Fukuoka 814-0175 Japan; 3 Center for Oral Diseases, Fukuoka Dental College, 3-2-1 Hakataekimae, Hakata-ku, Fukuoka City, Fukuoka 812-0011 Japan

**Keywords:** Osseointegration, Intraosseous stability, Insertion torque, Resonance frequency analysis, Voxel value, CBCT

## Abstract

**Background:**

A current implant body surface was treated with “rough processing” by sandblasting and acid etching for the purposes of obtaining more reliable osseointegration and shortening the treatment period. Various reports have examined the healing period with the use of these implant bodies, but a consensus opinion has not yet been obtained. The purpose of this study is to evaluate the relationship between insertion torque (IT) and implant stability quotient (ISQ) at implant treatment using the current rough-surfaced implant. We evaluated the implant treatment sites with ISQ values, IT values, and voxel values.

**Methods:**

Participants in this study comprised 26 patients (10 males, 16 females; mean age, 55.5 years) who received posterior region dental implants at Tokyo Dental College Hospital or Fukuoka Dental College Hospital. For all participants, pretreatment computed tomography and determination of bone quality from voxel values were performed. Thirty-two implant bodies were inserted into the posterior region, and insertion torque was measured. ISQ was also measured at 0, 2, 4, 6, 8, and 12 weeks postoperatively.

**Results:**

Eight implant bodies in the maxilla and 24 in the mandible were inserted. All ISQ values increased, exceeding 60 by 6 weeks postoperatively. For insertion torque < 30 N cm, ISQ increased significantly after 8 weeks. For ≥ 30 N cm, the ratio at which high ISQ values appeared increased significantly after 6 weeks. Compared with the treatment area with insertion torque < 40 N cm, the treatment area ≥ 40 N cm showed a significantly higher voxel value.

**Conclusions:**

No significant relationship was found between the insertion torque value and the ISQ value. Also, it was suggested that the ISQ value was considered to be an important indicator for observing the treatment state of the implant.

## Background

Dental implant treatments have improved in both convenience and predictability with refinements in implant bodies and treatment procedures as compared to about 50 years ago when clinical applications were started. Currently, an implant body surface is treated with “rough processing” by sandblasting and acid etching for the purposes of obtaining more reliable osseointegration and shortening treatment period. Despite previous reports about the healing period when implant bodies treated in this the procedure, a common consensus has yet to be obtained [1–3].

With implant treatment, the healing period refers to the period until an inserted implant body acquires osseointegration and can be loaded with occlusal force [4, 5]. In order to shave off the healing period, various method in which occlusal force was immediately or early loaded on the inserted implant body have been reported. However, theories on the therapeutic effect of immediate loading or early loading of implant treatment were not unified [6, 7]. “Quantity and quality of bone in treatment area,” “primary stability after implant insertion,” and “intraosseous stability during the healing period” are regional factors related to the acquisition and maintenance of osseointegration [8–10].

Usually, bone quantity and bone quality are evaluated by morphometry of computed tomography (CT) images and analysis of voxel values, and primary stability is evaluated as insertion torque (IT). Intraosseous stability of the implant during the healing period is estimated from X-ray images, the Periotest, or a resonance frequency analysis device [11–15]. The estimation procedure with a non-contact-type resonance frequency analysis device has been recognized as a non-invasive and reproducible procedure [16].

Intraosseous stability of an implant that is measured with a non-contact-type resonance frequency analysis device is evaluated as ISQ value. Insertion torque (IT) value and ISQ value are important indicators of implant treatment. However, the relationship between IT and ISQ is unclear. Some articles have reported positive correlations between IT and ISQ [15, 17], but others have found no correlation [18–20].

The purpose of this study is to evaluate the relationship between IT and ISQ at implant treatment using the current rough surfaced implant. We evaluated the implant treatment sites with implant stability quotient (ISQ) values, IT values, and voxel values. We assumed that there is relevance between the insertion torque value and the ISQ value.

## Methods

### Research design and study participants

This prospective study was conducted jointly by Tokyo Dental College (Tokyo, Japan) and Fukuoka Dental College (Fukuoka, Japan) from January to December 2015. All study protocols were conducted in accordance with the Declaration of Helsinki [21] and were approved by the ethics committees of Tokyo Dental College (approval #416) and Fukuoka Dental College (approval #213).

Participants comprised patients at Tokyo Dental College Hospital or Fukuoka Dental College Hospital who were ≥ 20 years old, desired implant treatment in the posterior region, and consented to the details of the study protocols. The participants of this study were selected without randomization.

In this study, implant treatment was performed on 33 tooth extracted sites. The reasons for tooth extraction were periodontal diseases (14), caries (12), root fractures (7), and teeth had already been extracted (8). Tooth extraction was carried out with normal technique without socket preservation method. All participants were followed up for more than 4 months after tooth extraction and X-ray examined with multi-slice CT (MSCT) or cone beam CT (CBCT). Consequently, it was confirmed that sufficient bone mass exists to insert the implant body without bone augmentation in all treatment site. CT imaging equipment was different for each facility.

Exclusion criteria were untreated systemic disease, diabetes, cardiovascular disease, osteoporosis, mental disorder, alcohol- or drug dependence, or smoking habit; failure to follow treatment directions; presence of severe periodontal disease, shedding disorder, trismus, malocclusion, or bruxism in the oral cavity; or failure of implant treatment.

### Materials and treatment procedure

**Fig. 1:**
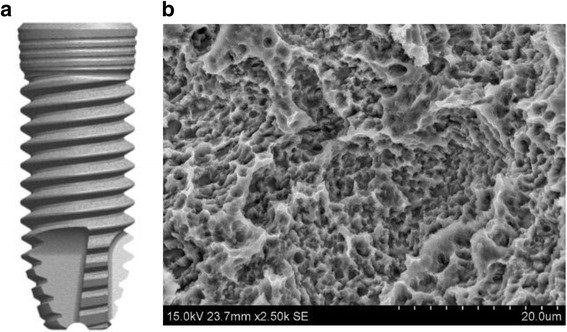
Genesio® Plus implant with Aanchor surface. Scheme of the dental implant body for the Genesio® Plus implants with Aanchor surface used. **a** Overview picture of Genesio® Plus implants with Aanchor surface. **b** Image from scanning electron microscopy. Both pictures were provided by GC Corporation. To obtain osseointegration from an early stage, the dental implant body was treated with sandblasting and acid etching from the neck to apex

The implant body used in this study was the Genesio® Plus implant with Aanchor surface (GC, Tokyo Japan). The implant body had been processed to create a rough surface by sandblasting and acid etching (Fig. 1). The implant body for the treatment of each participant was selected from two diameters (3.8 or 4.4 mm) and three lengths (8.0, 10.0, or 12.0 mm). The treatment area and the implant size used in this study are shown in Table 1.

**Table 1:** Treatment area and size of implant body

Number of implants	Treatment area (FDI)	Size of implant (mm)	
		Length	Diameter
1	14	10	3.8
2	14	10	3.8
3	14	10	3.8
4	16	8	3.8
5	16	10	4.4
6	16	8	4.4
7	16	8	4.4
8	17	10	4.4
9	36	10	3.8
10	36	10	4.4
11	36	8	3.8
12	36	10	3.8
13	36	12	4.4
14	36	10	4.4
15	36	10	4.4
16	36	12	3.8
17	36	10	4.4
18	36	12	4.4
19	36	12	4.4
20	37	10	4.4
21	37	12	4.4
22	37	12	4.4
23	46	10	4.4
24	46	10	4.4
25	46	10	3.8
26	46	8	3.8
27	46	12	4.4
28	46	10	3.8
29	46	12	4.4
30	47	10	3.8
31	47	8	3.8
32	47	10	4.4
33	47	10	4.4

The diameter of the implant body was selected to have a bone of 1 mm width or more in the around inserted implant body. The length of implant body was selected to leave 2 mm or more from the maxillary sinus or the mandibular canal

Implant treatment was performed in accordance with the procedure recommended by the manufacturer, without bone augmentation. A healing abutment was connected to the implant bodies after insertion (implant insertion in one stage method). A total of 17 dentists (treatment experience, 5–35 years; average, 11.5 years) performed all implant treatments in this study. All dentists who performed the implant treatment in this study were specialist certified by Japan Society of Oral Implantology and had experience of more than 5 years implant treatment.

### Evaluation of treatment

IT, ISQ, and voxel value were measured in this study. IT was measured immediately after the implant insertion using a torque wrench (GC, Tokyo, Japan).

ISQ was measured throughout the experimental period. To measure ISQ, a Smartpeg Type 21 (Osstell AB, Gothenburg, Sweden) was connected at 5 N cm to the implant body, measured using an Osstell ISQ™ (Osstell AB) three times from the buccal side. Average values were used for the evaluations. ISQ was measured immediately (0 week), and 4, 6, and 12 weeks after surgery in all cases, and also at 2 and 8 weeks after surgery where possible.

In the following cases, the implant body was excluded from the evaluation.

- If motion and/or rotation was observed in the implant body.
- If the bone surrounding the implant body showed absorption.
- If inflammation was observed in tissue surrounding the implant.
- If a mandatory ISQ measurement was not performed.

In this study, we performed X-ray image diagnosis using a multi-slice CT (MSCT) or a cone beam CT (CBCT) to confirm the healing of the bone form and volume after the tooth extraction. CT imaging equipment was different for each facility (two models of CBCT and one model of MSCT). It was difficult to make the same evaluation on voxel values obtained from different equipment. The X-ray examination performed in different two models of CBCT at 18 treatment sites and 8 treatment sites. Seven treatment sites were X-ray examined in MSCT. Therefore, bone quality was investigated at 18 treatment areas (8 in the maxilla, 10 in the mandible) on the CBCT performed under standardized conditions, as shown below.

**Fig. 2:**
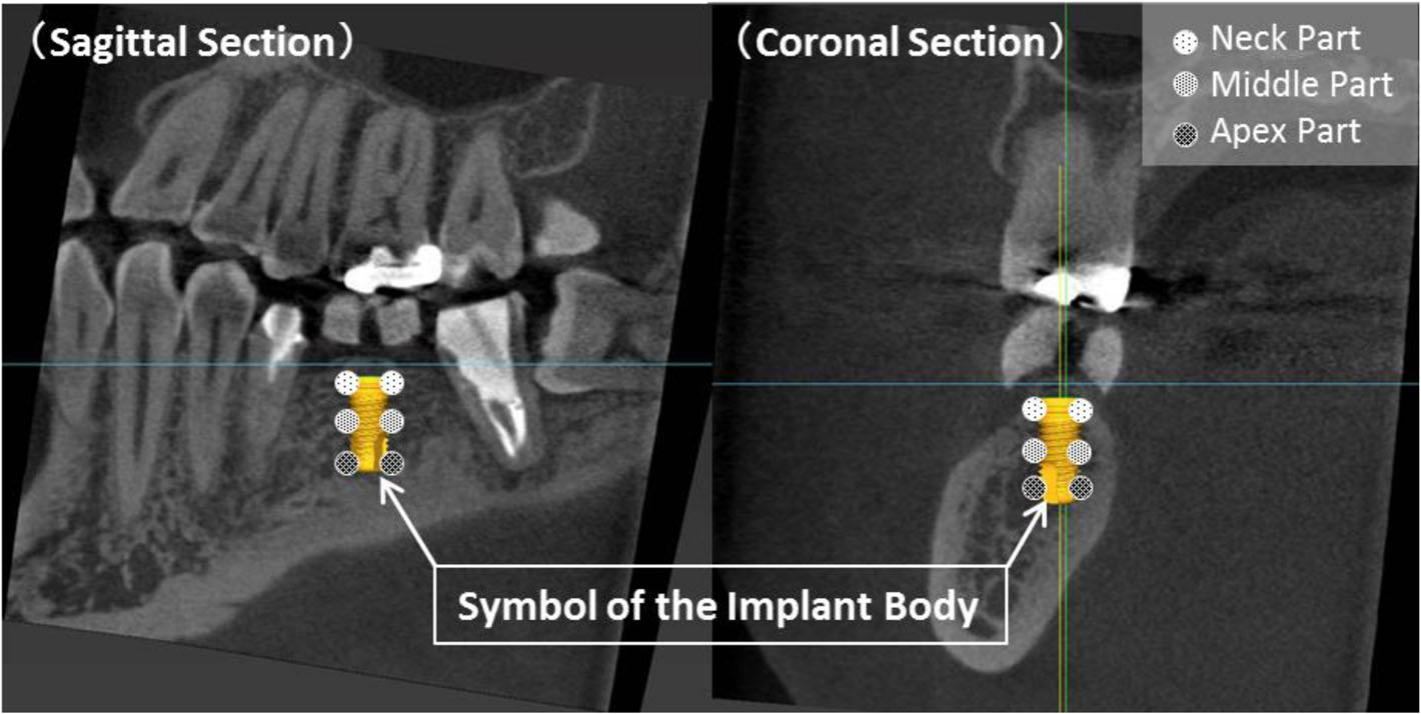
The measurement of the voxel values. A case of bone quality diagnosis before treatment. Width and height of the bone were measured to select the proper size of the implant body. The selected implant body was simulated on the bone images as a symbol, and then the voxel value was calculated as described in the “Methods” section

The CBCT was performed using a 3DX Multi-Image Micro CT FPD 8 system (J. MORITA MFG., Kyoto, Japan) (tube voltage, 80 kV; imaging area, 80 × 80 mm), and voxel values were measured with coDiagnostix™ 9.7 (dental wings, Montreal, Canada). The voxel values were calculated based on CT images for bone quality diagnosis. Voxel values were measured three times at 12 locations covering the mesial, distal, buccal, and lingual sides of each of the neck, middle, and apex parts of the implant treatment area, then average voxel values for each part and for the whole treatment area were calculated (Fig. 2).

### Statistical analysis

All numerical data obtained in this research were statistically analyzed with one-way analysis of variance and multiple comparison tests by Bonferroni et al. [22]. Fisher’s exact test and the corresponding *t* test were used for statistical analysis of insertion torque values and the relationship between ISQ and insertion torque, respectively [23].

## Results

### Study overview

A total of 33 implant bodies (8 in the maxilla, 25 in the mandible) were inserted into the 27 participants (11 men, 16 women), with the average age of 54.6 ± 12.2 years (range, 32–78 years). The average IT value was 32.7 ± 9.2 N cm (32.5 ± 11.6 N cm in the maxilla, 32.8 ± 8.5 N cm in the mandible). The diameter of the implant body was 4.4 mm in 20 (60.6%) and 3.8 mm in 13 (39.4%), and the length of the implant body was 8.0 mm in 6 (18.2%), 10.0 mm in 19 (57.6%), and 12.0 mm in 8 (24.2%) (Table 1).

The measurement results of IT and ISQ are shown in Table 2. Due to the identification of mobility at 4 weeks postoperatively, No. 19 implant body (diameter, 4.4 mm; length, 12.0 mm) that had been inserted at the first molar position in the right mandible of a 32-year-old male patient was excluded from the evaluation. As a result, this study evaluated the 32 implant bodies (8 in the maxilla, 24 in the mandible) inserted into 26 participants. The survival rate of the implant bodies at the end of this study was 97% (maxilla, 100%; mandible, 96%).

**Table 2:** Result of IT and ISQ

Number of implants	Insertion torque value (N cm)	Implant stability quotient value					
		0 week	2 weeks	4 weeks	6 weeks	8 weeks	12 weeks
1	25	33.0	75.0	77.0	78.3	79.7	77.0
2	40	68.0	70.3	70.0	72.0	75.7	75.3
3	40	78.3	77.0	78.0	78.7	80.0	80.0
4	35	74.0	43.0	61.0	73.0	75.7	80.0
5	45	85.3	85.7	84.0	83.3	84.0	83.0
6	25	65.0	68.0	68.3	70.0	72.0	71.0
7	40	82.3	84.0	82.0	84.0	81.0	81.0
8	10	51.3	52.1	46.7	59.3	66.3	68.7
9	35	78.7	80.0	80.0	80.0	81.3	80.0
10	35	86.3	84.0	83.0	84.0	85.0	85.0
11	40	78.0	75.7	76.3	78.7	79.7	80.0
12	40	80.0	80.0	79.3	81.0	81.0	84.3
13	35	71.0	73.0	77.3	80.0	77.0	82.0
14	45	70.0	72.0	76.0	80.0	81.0	82.3
15	30	73.3	73.0	65.0	68.7	73.3	75.0
16	25	75.3	75.0	72.0	73.0	75.3	77.7
17	35	68.7	76.0	72.7	76.3	79.0	81.3
18	30	75.0	80.0	80.3	81.7	82.7	81.3
19	30	50.7	–	–	–	–	–
20	30	73.7	61.3	67.7	73.7	57.7	57.0
21	45	69.3	66.7	57.3	75.0	76.3	77.7
22	20	73.3	75.7	76.7	77.0	80.7	82.0
23	50	71.7	69.7	73.3	76.0	77.3	79.7
24	30	76.0	80.0	80.0	82.0	81.3	81.0
25	20	64.0	47.7	71.0	69.3	68.0	77.0
26	20	65.0	70.0	75.0	77.0	79.0	80.0
27	35	65.7	60.3	61.3	69.7	68.0	68.0
28	40	22.3	57.0	68.7	75.0	75.0	80.0
29	35	69.7	73.7	76.7	74.7	74.7	81.0
30	20	58.0	46.0	65.7	70.0	70.0	75.3
31	35	67.0	78.0	69.3	78.7	80.0	82.3
32	20	53.3	63.3	61.0	64.7	68.7	70.0
33	40	52.0	52.7	66.0	75.7	78.0	79.3

The primary stability of no.19 implant was evaluated as good. ISQ of this implant was measured immediately after surgery. No.19 implant had mobility at 2 weeks after surgery and did not improve even at 4 weeks after surgery. Therefore, this implant was excluded from the evaluation

### Evaluation of ISQ and IT

**Fig. 3:**
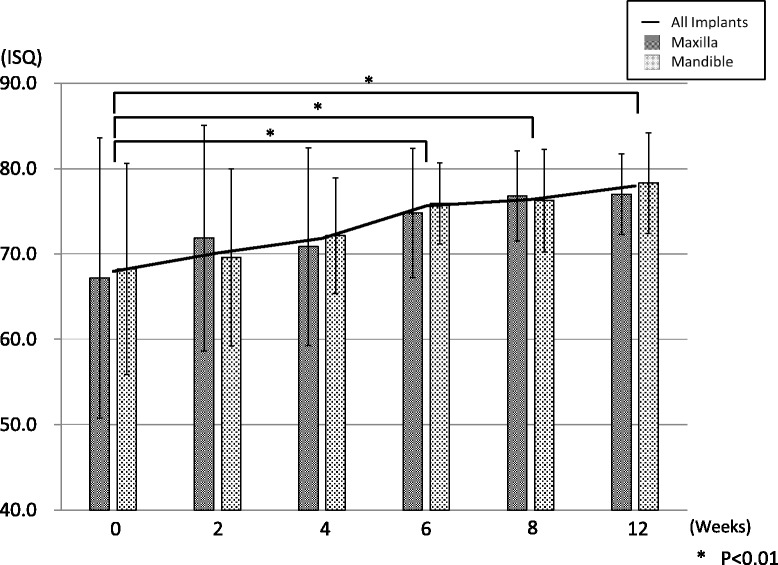
The evaluation of the average ISQ. Time-lapse migration of average ISQ. Average ISQ of all specimens increased in a time-dependent manner (results indicated by a line). A significant difference was observed by 6 weeks after surgery

The average ISQ values of all tested implant bodies increased through this study; moreover, all tested implant bodies indicated 60 or more ISQ value 6 weeks after the implant insertion. A significant difference was observed at 0 and ≥ 6 weeks (*P* < 0.01) (Fig. 3). No significant difference of average ISQ was found on the maxilla and mandible.

**Fig. 4:**
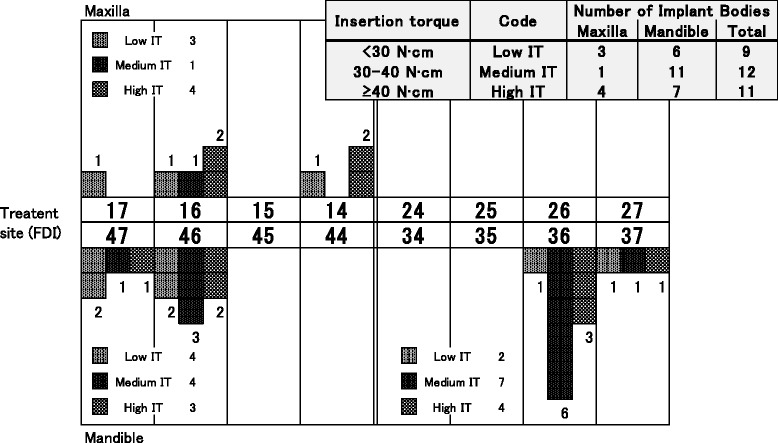
The classification of the insertion torque. All specimens classified into three groups according to insertion torque. Criteria for the classification are shown in the figure and in the “Methods” section

The inserted implant bodies were classified by IT value as the low IT group (< 30 N cm), the medium IT group (30–40 N cm), and the high IT group (≥ 40 N cm). Nine specimens were classified as the low IT group (3 in the maxilla, 6 in the mandible), 12 as the medium IT group (1 in the maxilla, 11 in the mandible), and 11 as the high IT group (4 in the maxilla, 7 in the mandible) (Fig. 4). There was no difference between the maxilla and the mandible in the average value of the IT (maxilla 32.5 ± 11.6 N cm, mandible 32.9 ± 8.7 N cm).

**Fig. 5:**
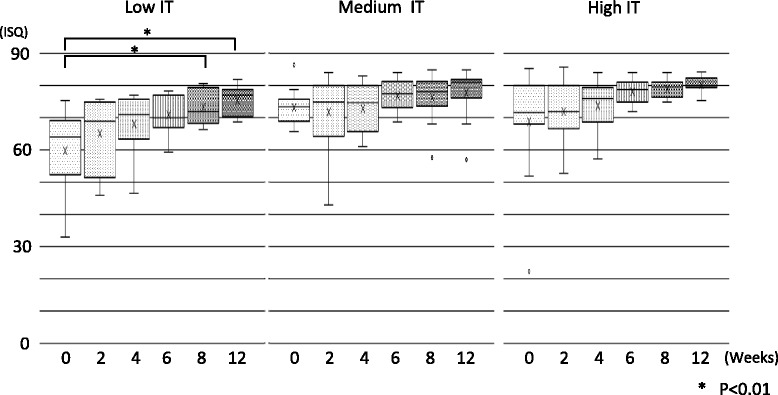
The comparison of ISQ values by the insertion torque. Time-lapse migration of ISQ values was compared with IT groups. Each IT group displayed similar migration. A significant difference in The ISQ was found in the low IT group after 8 weeks

Average ISQ tended to increase during the healing period in all IT groups (Fig. 5). Average ISQ of the low IT group was 59.81 at 0 week, increasing significantly after ≥ 8 weeks (*P* < 0.01). The average ISQ values of the medium and high IT groups at 0 week were 73.25 and 68.85, respectively. The average ISQ increased in a time-dependent manner at each group.

**Fig. 6:**
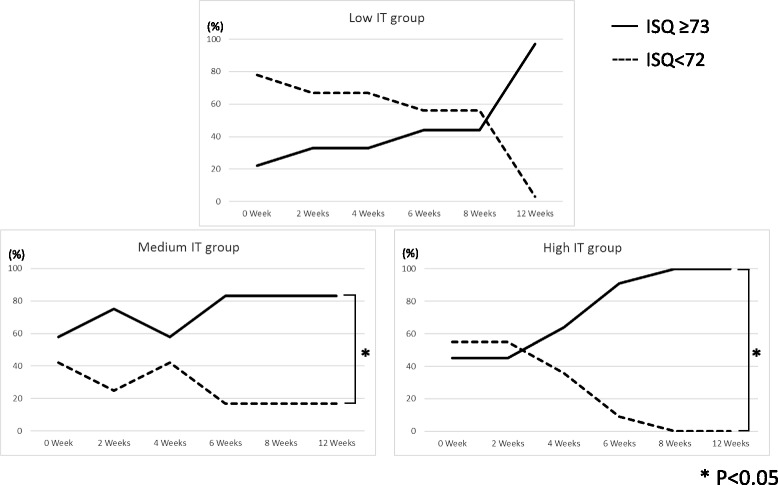
The relationship between ISQ and insertion torque. Percentage of specimens showing ISQ ≥ 73 compared with groups by week. In all groups, a period of rapidly increasing percentages was observed (8–12 weeks in the low IT group, 4–6 weeks in the medium and high IT groups). In the medium and high IT Group, a statistically significant difference was observed between ISQ ≥ 73 and ISQ < 72 (*P* < 0.05)

Total average ISQ in this study was 73.3 ± 9.6; therefore, an expedient reference value was defined as the ISQ 73, then the percentage of specimens showing ISQ ≥ 73 was determined (Fig. 6). ISQ ≥ 73 was observed from 8 to 12 weeks in the low IT group and from 4 to 6 weeks in the medium and high IT groups. Significant differences in the incidence of ISQ ≥ 73 was recognized at the medium and high IT groups (corresponding *t* test, *P* < 0.05).

### Relationship between IT and voxel value

In this study, conditions and environments for CT imaging in each facility were different. Bone quality was investigated at 18 treatment areas (8 in the maxilla, 10 in the mandible) on CBCT performed under standardized conditions, as shown below. CBCT was performed using a 3DX Multi-Image Micro CT FPD 8 system (J. MORITA MFG., Kyoto, Japan) (tube voltage, 80 kV; imaging area, 80 × 80 mm), and voxel values were measured with coDiagnostix™ 9.7 (dental wings, Montreal, Canada).

**Fig. 7:**
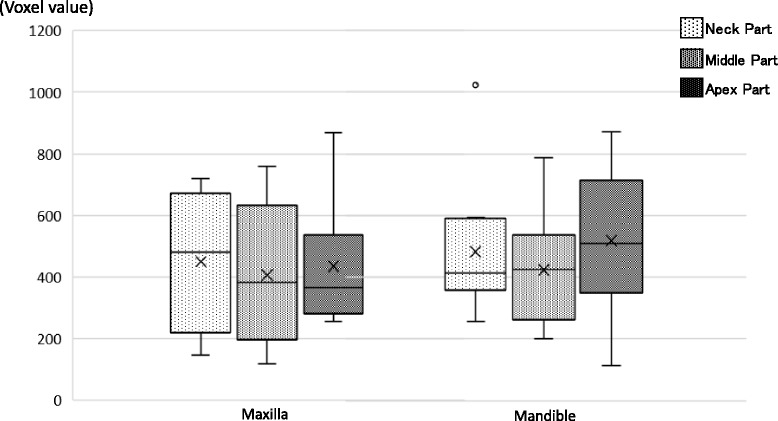
The average voxel value between the maxilla and mandible. There was no difference between the maxilla (430.9 ± 211.6) and the mandible (475.6 ± 211.5) in the average voxel value. Also, no difference was found in each part

**Fig. 8:**
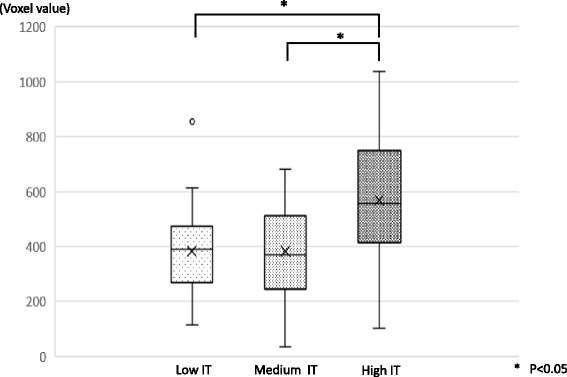
The relationship between average voxel value and insertion torque (averaged over the entire treatment area). The comparison of average voxel value among IT groups. Average voxel value was 384.0 ± 154.6 in the low IT group, 387.7 ± 147.7 in the medium IT group, and 619.2 ± 200.4 in the high IT group

**Fig. 9:**
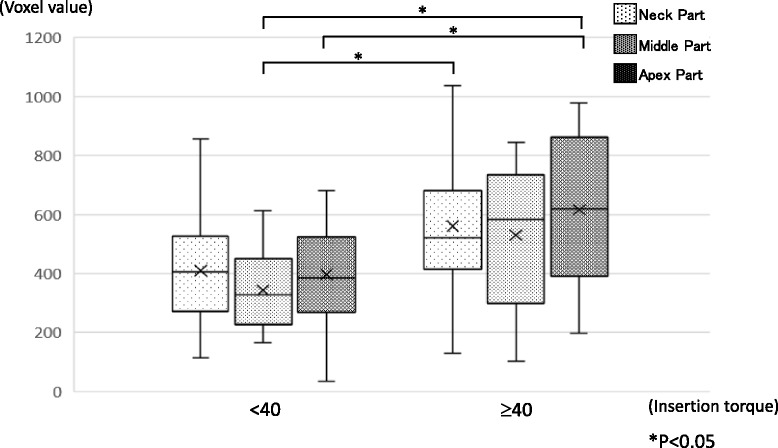
The comparison of two groups at average voxel values for each part. The comparison of voxel values by insertion torque. All specimens were classified into two groups by insertion torque < 40 and ≥ 40. The < 40 group represents a combination of the low and medium IT groups

There was no difference of average voxel value between the maxilla and the mandible (Fig. 7). In comparison between average voxel values and IT groups, the low and medium IT groups showed no significant differences, but the high IT group showed voxel values ≥ 40% higher than the other groups (Fig. 8). A significant difference was observed between the low/medium IT groups and the high IT group (*P* < 0.05). A significant difference was observed between the low/medium IT groups and the high IT group (*P* < 0.05). Also, voxel values at each part of the implant (neck, middle apex) were compared with IT < 40 and ≥ 40. The results suggested that the neck and apex parts in the ≥ 40 IT group showed significantly higher voxel values than the middle and apex parts of the < 40 IT group (*P* < 0.05) (Fig. 9).

## Discussion

According to the previous literature, the obtaining osseointegration is integral to the intraosseous stability of the implant body during the healing period [24]; moreover, the importance of postoperative assessment of the intraosseous stability of the implant has also been reported [10]. Intraosseous stability of the implant body is evaluated immediately after the implant insertion and during the healing period after surgery.

The primary stability is necessary for implant treatment, and the absence of primary stability may result in treatment failure [25, 26]. Primary stability was evaluated with insertion torque immediately after the implant insertion.

This study had a short implementation period, and it was difficult to recruit a large number of participants. Therefore, participants had a bias in age and gender, and the treated area was also biased. We referred previous publication to review the effect of participant’s age and gender at insertion torque value. According to the above literature reviewing, the participant’s age and gender did not affect insertion torque value [27–29]. However, it was thought that there was a possibility that the number of participants influenced the result.

In this study, primary stability was evaluated with insertion torque value measured by manual torque wrench immediately after implant insertion. Manual torque wrench is the medical instrument used for implant treatment frequently. A recent study about insertion torque that compared electronically controlled torque wrench with manual torque wrench states that the measurement results of both instruments were similar [30]. Manual torque wrench is classified into three styles (coil, toggle, and beam style). In this study, beam style manual torque wrench was used. It was reported that beam style torque wrench present most precise result compared with other two kinds of torque wrenches [31]. As described above, the measurement procedure of insertion torque value in this study is thought to be acceptable.

The insertion torque value in this study showed broader (10 to 50 N cm) than the previous publication (Table 2) [22, 32], and the cause of reasons for the difference are as follows: Primary stability may be affected by the bone quantity and bone quality in the treatment area, the micro- and macro-level design of the implant body, and the accuracy of the surgical technique [18, 25]. In this study, the 17 dentists performed implant treatment. The deviation of each insertion torque value was thought by the surgical technique of each dentist. In clinical situation, the insertion torque value is considered to indicate various values.

The insertion torque value in this study showed no significant difference between each treatment area. Therefore, all of the implant bodies were considered as one population and that population was classified into three groups by insertion torque value and analyzed. In a recent literature, Anitua et al. reported that the insertion torque values were 59.29 ± 7.27 N cm at type I bone, 56.51 ± 1.62 N cm at type II bone, 46.40 ± 1.60 N cm at type III bone, 34.84 ± 2.38 N cm at type IV bone, and 5 N cm at type V bone [29]. Since the average value of insertion torque in this study was 32.7 ± 9.2 N cm, it was inferred that this study evaluated implant treatment for relatively soft bone quality.

The intraosseous stability of the healing period was evaluated by mobility measurement and/or resonance frequency analysis. A resonance frequency analysis has been reported as a non-invasive procedure that is useful for evaluating osseointegration [13, 33]. The results of the resonance frequency analysis were represented in the present study as the ISQ.

An ISQ is reportedly affected by the condition of the bone surrounding the implant, such as the range of contact between implant body and bone [33–35]. Other studies have suggested that ISQ immediately after implant insertion should be about 60 [24, 36], with ISQ subsequently decreasing over weeks 0–4 and increasing over weeks 4–8 after surgery [13, 24, 34]. ISQ values 57–70 may indicate that intraosseous stability of the implant body is constant [34, 37].

Increases or decreases of ISQ values are explained as follows: The inserted dental implant body is supported by mechanical interdigitating force after surgery, but this interdigitating force will be reduced time-dependently by the effects of osteoclasts activation at the initial stage of the bone remodeling process, then osseointegration will be completed by an increasing contact area between the bone and dental implant body at the bone regeneration step [38]. The period switch from ISQ decreasing to increasing was considered as the most unstable but important period during the healing period [24].

The average ISQ in this study was 68.0 ± 13.7 after surgery then increased to 71.8 ± 8.3 at 4 weeks and 78.0 ± 5.7 at 12 weeks after surgery; all inserted implants showed ISQ > 60 after 6 weeks (Fig. 3). In addition, the average ISQ decreasing was not observed during the experimental period. According to the publication about the relationship with ISQ value and intraosseous stability of the implant body inserted in the soft bone quality by Held et al., the ISQ value was not decreasing and tended to increase [39]. As per we evaluated implant treatment at the soft bone in this study, migration of the ISQ value in this study showed similarity with the abovementioned document.

The relationship between IT and ISQ remains unclear. Some articles have reported positive correlations between IT and ISQ [15, 17], but others have found no correlation [18–20].

While no significant relationship was found between IT and ISQ in this study, the migration pattern of ISQ differed between the low IT group and medium/high IT group. ISQ in the low IT group was initially low, increasing over time. A significant difference was observed between 0 and ≥ 8 weeks (Fig. 5). The ISQ did not change significantly during the experimental period in the medium or high IT groups, but the percentage of high ISQ (≥ 73) specimens was significantly higher at 4 to 6 weeks compared to other time periods in both groups (Figs. 5 and 6). The results in this study suggest that if the implant insertion has been performed with low insertion torque, progress of peri-implant bone maturation (transfer from mechanical interdigitation to osseointegration) slowly stabilizing at 8 weeks after surgery, or if the insertion torque value was moderate or higher, peri-implant bone will maturate following a safe healing period and show stabilization at 6 weeks after surgery.

In this study, we could not find a significant relationship between insertion torque value and ISQ value. However, insertion torque value is an important indicator for predicting the progress of implant treatment, and ISQ value is considered to be an important indicator for observing the treatment state of the implant. Currently, the insertion torque value is used as the major decision index for the determination of the loading period on the implant body. Lozano-Carrascal et al. explained that if the insertion torque value shows between 32 to 50 N cm, the implant treatment with immediate loading protocol is able to apply [40]. Also, Anitua et al. applied immediate loading protocol when the insertion torque value was 40 to 65 N cm (the average insertion torque value is 55 ± 3.48 N cm) [29]. As described above, the insertion torque value used as a decision index of the loading period of the implant is yet undefined. Therefore, the loading period of the implant should not be determined immediately after insertion but should be determined after careful follow-up observation. When deciding to load period, the ISQ value will be an important decision index.

A bone quality of the treated area may affect primary stability as described above, preoperative analysis of bone quality is important for clarifying the primary stability of dental implants. This study analyzed bone quality using voxel values obtained using Digital Imaging and Communications in Medicine (DICOM) data from CBCT. According to the result of that analysis, it was suggested that insertion torque is high when inserting the implant body used in this study into the bone with high voxel value (Fig. 7). Moreover, in this study, the ISQ values of implant bodies showing insertion torque of 30 or more N cm were stabilized at a high value (ISQ was 73 or more) in 6 weeks after insertion (Figs. 5 and 6). These results may suggest that if the implant body used in this study is inserted into the bone of sufficient quality, high IT then intraosseous stability during the healing period can be expected, and osseointegration may be completed by 6 weeks after surgery. In addition, in order to judge the completion of osseointegration, an evaluation of intraosseous stability in the healing process after insertion of the implant body is necessary. There was a possibility that the implant body used in this study could be treated with the early loading method [1, 2]. In order to make this result more reliable evidence, it seems necessary to conduct a randomized controlled study on more participants.

As accurate CT attenuation was not measured due to the lower spatial resolution of CBCT compared with MSCT, a CBCT was recognized as unsuitable for evaluating bone quality. However, several groups have recently reported the potential use of CBCT systems as an apparatus for estimating bone quality. Isoda et al. described a high correlation between voxel values obtained by CBCT and IT of the implant [41]. Moreover, Nomura et al. reported a high correlation between density values from the CBCT and MSCT systems [42].

According to the measurement of the average voxel values in this study, a significant difference was seen between the high IT group and the low/medium IT group, but no significant difference was found between the low and medium IT groups (Fig. 7). Specimens showing IT ≥ 40 N cm were thought to have a good bone quality, and voxel values at each part of the implant (neck, middle apex) were compared between groups with IT < 40 (combined low and medium IT groups) and ≥ 40 (high IT group) (Fig. 8). The results suggested that the neck and apex parts in the high IT group showed significantly higher voxel values than the middle and apex parts of the low/medium IT group.

Using a MSCT system for preoperative diagnosis of bone quality, classified as five stages according to CT attenuation, and detailed diagnosis was performed for the whole treatment area [43]. In this study, no significant difference was found when bone quality was compared between the three different IT groups, but when comparisons were made between two groups (low/medium vs high), significant differences were observed between groups and also between measurement sites (Figs. 6 and 7). Diagnosis of bone quality using CBCT does not seem as detailed as results from MSCT, but the diagnosis of whether bone quality is sufficient appears feasible.

CBCT systems offer many advantages over MSCT systems, including low exposure doses, high resolution, reduction of metal artifacts, ease of installation, and utility as a diagnostic tool in implant treatment [41, 42]. Due to the expanded utility of CBCT systems for dental implant diagnosis, the establishment of techniques for diagnosing bone quality by CBCT is necessary.

## Conclusions

The purpose of this study was to evaluate the relationship between the insertion torque value and the ISQ value at the implant treatment using the current rough-surfaced implant. As a result, no significant relationship was found between the insertion torque value and the ISQ value. Also, it was suggested that the ISQ value was considered to be an important indicator for observing the treatment state of the implant. In addition, it was considered that there is a possibility that the early loading protocol can be applied to the implant body used in this study.

## Abbreviations

CBCT: Cone beam CT
CT: Computed tomography
DICOM: Digital Imaging and Communications in Medicine
ISQ: Implant stability quotient
IT: Insertion torque
MSCT: Multi-slice CT
